# Canadian packaged gluten-free foods are less nutritious than their regular gluten-containing counterparts

**DOI:** 10.7717/peerj.5875

**Published:** 2018-11-06

**Authors:** Jennifer A. Jamieson, Mary Weir, Laura Gougeon

**Affiliations:** Department of Human Nutrition, St. Francis Xavier University, Antigonish, Nova Scotia, Canada

**Keywords:** Gluten-free, Iron, Folate, Diet, Nutrient, Celiac, Fibre, Gluten, Protein

## Abstract

**Background:**

A strict gluten-free (GF) diet is required for the management of celiac disease (CD). The nutritional adequacy of this diet has been questioned due to the elimination of wheat, an important vehicle for micronutrient fortification and source of fibre. While novel and/or reformulated packaged GF products have rapidly entered the marketplace, providing alternatives to wheat-based staples, it is unknown whether these new products are nutritionally comparable.

**Methods:**

From a database of 3,851 foods collected across 21 grocery stores in Eastern Canada, we compared the nutrient content of 398 unique GF items with 445 gluten-containing (GC) equivalents. Wilcoxon rank tests were conducted on listed nutrient content (g, mg, µg) per 100 g of product and the nutrient contribution of iron, folate and fibre were evaluated using Health Canada’s nutrient claim regulations.

**Results:**

GF staples (cereals, breads, flours, pastas) contained 1.3 times more fat and less iron (by 55%), folate (by 44%) and protein (by 36%), than GC counterparts (*P* < 0.0001). On average, GF pastas had only 37% of the fibre in GC pastas (*P* < 0.0001). Notably, GF and GC flours were equivalent in nutrient content. Despite GF and GC flours having similar nutritional content, the vast majority of the processed GF foods fell short in key nutrients.

**Discussion:**

Packaged GF foods in Canada are generally less nutritious than their GC counterparts, suggesting that GF diets should not be promoted to those who do not require it. The use of nutrient-dense GF flours in homemade foods may improve nutrient intakes on the GF diet.

## Introduction

Following a gluten-free (GF) diet is the only treatment option currently available for individuals with celiac disease (CD) and other gluten-related disorders such as gluten ataxia and wheat allergy (Elli et al., 2015;  Hadjivassiliou, Sanders & Aeschlimann, 2015). CD is a heritable, autoimmune disease that is estimated to affect approximately 1% of the population worldwide (Elli et al., 2015). Although less well studied, the emergence of non-celiac gluten sensitivity (NCGS) or non-celiac wheat sensitivity (NCWS)—in which gluten and/or wheat consumption triggers clinical CD symptoms, such as bloating, abdominal discomfort, and diarrhea, but does not lead to the gastrointestinal tract damage and immune reactions characteristic of CD—has led to wheat/gluten restriction in this patient group (Lovik et al., 2017). Although the pathogenesis of NCGS or NCWS is unclear, present knowledge suggests it is not autoimmune and not allergy-related (Elli et al., 2015). Perceived gluten intolerance has also been reported in 42% of patients with Irritable Bowel Syndrome (Aziz et al., 2015) and symptomatic improvement was observed with four weeks of gluten restriction in a controlled trial with IBS patients (Zanwar et al., 2016). Finally, a recent survey showed that almost 29% of Canadians are looking for GF products, but only 7% (1% for CD; 6% for gluten intolerance) of them do so for medical reasons (Agriculture and Agri-Food Canada, 2014). The remaining 22% either perceived GF to be a healthier choice or may have had a family member requiring gluten avoidance. Thus, the nutritional quality of the GF diet is an important consideration for growing patient populations, as well as the general public.

Gluten restriction has important implications for nutrient adequacy, since staple western foods (e.g., breads, pastas, and cereals) are key nutrient sources in the western diet, especially when consuming whole grain foods (Dietitians of Canada, 2018). Wheat bran, in particular, is a rich source of insoluble fibre, phytochemicals and micronutrients (Stevenson et al., 2012). In Canada and the US, the fortification of wheat flour with folic acid, iron, thiamin, riboflavin, and niacin is mandatory (Government of Canada, 2018; US Department of Health and Human Services, 2015). These regulations do not apply, however, to alternative GF flours (e.g., rice, tapioca, chickpea). GF product reformulations, may require additional fat and/or sugar to improve organoleptic characteristics, simultaneously increasing energy density (Gallagher, Gormley & Arendt, 2004). The replacement of wheat with white rice or refined starches, may further lower nutrient density. Thus, depending on the overall dietary pattern, nutrient inadequacy and weight management may be long-term concerns for GF diets.

In people diagnosed with CD, iron deficiency and anemia, inadequacy of B vitamins such as folate, zinc and calcium, and low bone mineral density are common concerns (Silvester & Rashid, 2007). Diet assessment studies with CD patients following long-term GFD, have reported inadequate intakes of fibre, iron, calcium, and B vitamins in Australia (Shepherd & Gibson, 2013), Britain (Wild et al., 2010), and Germany (Martin et al., 2013). Several studies have surveyed the nutrient content of gluten-free foods available for purchase around the world (Estevez et al., 2016; Missbach et al., 2015; Wu et al., 2015) but vary according to national nutrient fortification regulations, sampling methods used and sample size. In Canada, a 2012 grocery store survey across three large cities identified only 71 GF-labelled foods and reported a 168% higher price, on average, for GF versus GC items (Kulai & Rashid, 2014). Due to small sample sizes per food category (<10), nutrient comparisons were limited. However, over 2,300 new foods products were marketed as GF in Canada between 2007 and 2013, when these specialty products grew from a market share of 4.5% to 15.4% (Agriculture and Agri-Food Canada, 2014). To capture this surge in GF product availability and its expected changes in pricing, we developed a database containing nutrient and price information of 2,226 GF-labelled foods and 1,625 regular comparison products sold in non-urban grocery stores (*n* = 21) of the Maritime Provinces (Jamieson & Gougeon, 2017). Thus, using this database and a study design similar to a case-control, we investigated whether nutrient content of non-duplicate GF foods differ from their regular GC counterparts. Differences in price and availability of GF and GC items were reported previously (Jamieson & Gougeon, 2017).

## Materials & Methods

### Store selection

Overall, 21 grocery stores were visited between August, 2014, and August, 2015 in Nova Scotia (*n* = 16), Prince Edward Island (*n* = 3) and New Brunswick (*n* = 2). These stores were selected based on convenience, population size (rural population <4,000 or town population 4,000–10,000), and distance from large urban centres (rural stores ≥40 km from a town store). Four stores were located in towns, while 17 stores were in rural areas. Eighteen stores were part of three national grocery retailers, and three were independently-run grocers. Specialty health food stores were excluded from sampling, as these stores were not common in the rural areas where this study was conducted.

### Product selection

All GF-labelled food products on display were noted on one (or more) visits to each grocery store. Naturally GF foods (e.g., rice, meat, fruits, vegetables) and foods without nutrition fact panels were not included. For each GF product found, a research assistant recorded the brand, product description, price, and portion size (weight or volume) and took a photograph of the nutrition facts panel and the package front. The listed (i.e., regular) product price was noted, regardless of the product being on sale at the time of observation. For every GF product, the same information was recorded for a comparable gluten-containing (GC) product, preferably from the same manufacturer, at the same visit. The assistant followed these general crietria when selecting a comparable GC product: (1) a product whose description most closely matched that for the GF product was chosen; (2) in the case of multiple GC alternatives being available, a recognizable brand was chosen, instead of store owned-brands, for example; and (3) to increase representativeness of the GC sample, in the case of multiple GC alternatives being available for a previously sampled GF product, the assistant chose a comparable GC product not sampled in previous visits. In some cases, however, the same GC counterpart had to be selected to match several GF products, as it was the only similar GC product available. The final database contained 2,454 GF products and 1,822 GC matches. Nineteen products that reported servings in volume (mL) were converted to weight (g) using an online density calculator, while 425 volumetric (non-staple) products were excluded, as described elsewhere (Jamieson & Gougeon, 2017).

### Data entry

All products were categorized as either ‘staple grain-based foods’ or ‘non-staples’, with other sub-categories adapted from previous research (Kulai & Rashid, 2014). Staples included breads, flours, cereals, and pastas. Details and descriptions of these food categories were previously reported (Jamieson & Gougeon, 2017). In the present study, muffins, sweet loaves and waffles were considered ‘quick breads’, and the ‘cereal bar’ category included any foods labelled as cereal, granola, or energy bar. In the staple food category, a sub-category of ‘whole grains’ was created to accommodate items that had a whole grain (e.g., whole wheat, oat, barley, brown rice, quinoa, buckwheat, multigrain or flours of these grains) as the first ingredient, as well as a whole food grain alternative (e.g., almond flour, chickpea flour). If the first ingredient was not a whole grain (i.e., white flour, white rice, processed starches, or corn), the food was classified as ‘non-whole grain’. Although corn is technically a grain, we considered it a non-whole grain in this study due to high starch content and low nutrient density.

### Data analysis

For this study, unique, non-duplicate GF products (i.e., products that did not have the same name, brand, weight and manufacturer collected from multiple stores) were treated as “cases”, while the unique, non-duplicate GC counterparts were treated as “controls”—similarly to a case-control in observational study designs. Approximately 52.5% of identified food products in the database were duplicate items and, hence, excluded from analysis. Data are reported in median nutrient content (µg, mg, or g) per 100 g of GF versus GC products and compared using Wilcoxon rank sum test (alpha-level = 0.05) due to non-normality. Proportions were compared using *χ*^2^ tests. Due to smaller sample sizes folate content was analyzed for all staple foods but not separately by food category. Fibre, iron and folate contents of GF and GC foods were compared to Health Canada’s nutrient claim criteria for these nutrients (Canadian Food Inspection Agency, 2018) to develop consumer-friendly practical applications from this work. According to these criteria, fibre content is evaluated by grams per serving as follows: not a source, <2 g; source of fibre, ≥2 g; high source, ≥4 g; or very high source, ≥6 g. Iron and folate are evaluated by the total nutrient content as follows: not a source, <5%; source, ≥5%; good source, ≥15%; very good/excellent source, ≥25% of the recommended daily intake (RDI) (RDI = 14 mg iron; 220 µg folate). All analyses were done in StataSE 14.2 (StataCorp, 2015. College Station, TX, USA).

## Results

### Sample description

From the GF product database, 398 unique GF products from eight grain and/or cereal-based food categories were compared to 445 GC items. No significant differences were observed in the proportions of ‘whole grain’ versus ‘non-whole grain’ products overall (44.1%, GF; 49.1% GC; *χ*^2^ *P* = 0.301), or among the staple food category.

### Staple foods

Overall, GF staple products contained 1.3 times more fat per 100 g of product, as well as 36% less protein, 55% less iron , and 44% less folate, than GC counterparts (Table 1). GC whole-grain staple items had 1.5 times more fibre, on average, than GF whole-grain items (*P* < 0.0001). On the other hand, mean fibre was 1.2 fold higher in GF non-whole grain items than in GC non-whole grain items (*P* = 0.0400).

**Table 1:** Differences in nutrient content between staple (breads, flours, cereals and pasta) gluten-free and gluten-containing foods per 100 g of product, according to whole grain categorization.

	**All staples, median (IQR)**			**Whole grain staples, median (IQR)**			**Non-whole grain staples, median (IQR)**		
Nutrient per 100 g	Gluten-free (*n* = 202)	Regular (*n* = 220)	*P* value	Gluten-free (89)	Regular (*n* = 108)	*P* value	Gluten-free (*n* = 113)	Regular (*n* = 112)	*P* value
Calories (kcal)	357 (286–376)	353 (269–367)	0.1976	365 (326–400)	353 (290–385)	0.0777	346 (283–365)	326 (266–367)	0.6133
Fat (g)	3.6 (2.0–7.8)	2.8 (1.8–5.0)	0.0001	3.8 (2.9–10.3)	3.6 (1.8–5.6)	0.0032	3.1 (1.5–6.7)	2.2 (1.2–3.3)	0.0032
Saturated fat (g)	0.5 (0.0–1.1)	0.5 (0.2–0.9)	0.7890	0.7 (0.4–1.2)	0.7 (0.4–1.0)	0.3461	0.4 (0.0–0.9)	0.4 (0.2–0.7)	0.2444
Carbohydrate (g)	72.9 (48.3–78.9)	70.6 (50.0–74.1)	0.1235	73.3 (48.8–78.3)	72.5 (48.3–77.6)	0.6260	72.9 (48.6–80.0)	61.7 (49.3–74.1)	0.1424
Fibre (g)	3.6 (2.4–7.5)	4.8 (2.7–9.4)	0.0688	6.5 (3.4–9.5)	9.4 (6.7–10.7)	<0.0001	3.3 (2.1–5.7)	2.8 (2.0–3.5)	0.0400
Sugars (g)	3.4 (0.0–10)	3.5 (2.4–7.0)	0.1464	2.9 (0.0–14.3)	4.3 (2.4–17.3)	0.0575	4.0 (0.0–8.8)	3.5 (2.4–4.9)	0.9206
Protein (g)	7.0 (5.3–8.5)	10.9 (8.6–13)	<0.0001	7.6 (6.7–9.5)	11.4 (9.3–13.3)	<0.0001	5.9 (4.5–7.1)	9.3 (7.8–12.9)	<0.0001
Sodium (mg)	118 (0.0-442)	317(5.3–415)	0.1687	50 (0–403)	202 (5.9–379)	0.3160	339 (0.0–455)	354 (4.70–515)	0.2176
Iron (mg)	1.5 (0.8–2.8)	3.3 (2.9–4.7)	<0.0001	2.2 (1.1–3.0)	3.5 (2.6–11.1)	<0.0001	1.0 (0.6–2.4)	3.3 (3.1–4.7)	<0.0001
Folate^a^ (µg)	35.5 (11.1–58.7)	62.9 (51.8–207)	<0.0001	23.4 (9.8–42.3)	60.0 (50.3–65.2)	<0.0001	52.0 (25.7–64.0)	147 (58.7–207)	<0.0001

**Notes.**

IQR interquartile range

a For folate, *n* = 43 gluten-free staples and *n* = 127 regular staples; whole grain *n* = 17 gluten-free and *n* = 63 regular; non-whole grain *n* = 26 gluten-free and *n* = 64 regular.

Within the staple food categories, fat content was higher in GF versus GC breads and pastas, and protein content was lower in GF versus GC breads, crackers, cereals and pastas (Table 2). GF breads had more sugar and less carbohydrate (starch) content than GC breads. Conversely, GF crackers and pasta had less sugar and more carbohydrate content than GC versions. Fibre content was similar in both GF and GC items, except within the pasta category, with GC items having 2.7-fold more fibre. Amongst breads, crackers, cereals and pastas, iron content ranged from 3 to 4.5-fold lower in GF items compared to GC counterparts. No nutritional differences per 100 g of GF and GC flours were noted, with the exception of higher sodium content in GF than GC flours (Table 2).

**Table 2:** Differences in nutrient content between gluten-free and gluten-containing staple foods per 100 g of product, according to bread, flour, cereal and pasta sub-categorization.

	Bread, median (IQR)			Flours, median (IQR)		
Nutrient per 100 g	Gluten-free (*n* = 72)	Regular (*n* = 87)	*P* value	Gluten-free (*n* = 25)	Regular (*n* = 22)	*P* value
Calories (kcal)	268 (233–290)	266 (250–280)	0.9876	354 (350–400)	367 (367–367)	0.7910
Fat (g)	6.8 (4.8–9.1)	3.3 (2.5–4.9)	<0.0001	1.5 (0.0–5.0)	1.3 (0.5–1.7)	0.4498
Saturated fat (g)	0.7 (0.4–1.3)	0.7 (0.5–0.9)	0.3205	0.0 (0.0–0.5)	0.3 (0.0–0.3)	0.3534
Carbohydrate (g)	44.9 (41.7–49.5)	48.0 (45.3–52.2)	0.0018	76.7 (64.3–80.0)	73.3 (73.3–73.3)	0.2770
Fibre (g)	3.4 (2.3–6.6)	2.7 (2.1–5.0)	0.0676	8.0 (2.5–16.7)	3.3 (3.3–10)	0.9583
Sugars (g)	5.1 (2.9–7.0)	4.0 (2.5–5.4)	0.0193	0 (0.0–3.0)	0 (0.0–3.3)	0.6470
Protein (g)	5.0 (3.9–7.2)	8.9 (8.0–10)	<0.0001	8.2 (5.0–16.7)	13.3 (13.3–13.3)	0.0815
Sodium (mg)	449 (358–539)	393 (353–476)	0.0326	4.0 (0–29)	0 (0–0)	0.0027
Iron (mg)	1.0 (0.6–2.2)	3.1 (2.6–3.3)	<0.0001	3.9 (0.9–6.5)	4.7 (4.7–4.7)	0.3986

	Cereals, median (IQR)			Pastas, median (IQR)		
Nutrient per 100 g	Gluten-free (*n* = 52)	Regular (*n* = 60)	*P* value	Gluten-free (*n* = 43)	Regular (*n* = 50)	*P* value
Calories (kcal)	400 (375–436)	385 (367–400)	0.1058	357 (353–365)	353 (353–365)	0.0025
Fat (g)	3.4 (2.5–12.9)	5.0 (1.8–6.7)	0.7656	2.4 (1.2–2.7)	1.8 (1.2–1.8)	0.0018
Saturated fat (g)	0.7 (0.0–2.1)	0.9 (0.0–1.5)	0.8796	0.4 (0.0–0.6)	0.4 (0.2–0.5)	0.7795
Carbohydrate (g)	80.0 (69.2–84.8)	78.2 (70.5–83.3)	0.5971	77.6 (76.8–78.8)	72.9 (71.8–74.1)	<0.0001
Fibre (g)	7.5 (4.3–10)	8.8 (4.6–11)	0.3820	3.5 (2.4–3.6)	9.4 (3.5–9.4)	<0.0001
Sugars (g)	20.0 (8.6–23.1)	17.4 (5.2–22.3)	0.4345	0.0 (0.0–0.0)	2.7 (2.4–3.5)	<0.0001
Protein (g)	7.1 (6.7–10)	9.3 (6.9–11.9)	0.0203	7.1 (5.9–7.8)	12.9 (12.9–14.1)	<0.0001
Sodium (mg)	117 (31.1–417)	352 (109–615)	0.0106	0.0 (0.0–0.0)	2.4 (0.0–5.9)	0.0002
Iron (mg)	2.8 (1.9–4.2)	12.7 (4.2–14)	<0.0001	1.0 (0.49–1.5)	3.29 (3.29–3.29)	<0.0001

**Notes.**

IQR interquartile range

### Non-staple grain or cereal based products

Non-staple GF and GC food products did not differ in energy or carbohydrate content (Table 3). GF cereal bars were 1.5-fold higher in fat than GC bars, whereas GF cookies were 1.5-fold higher in saturated fat than GC cookies. GF cereal bars were 1.3-fold higher in fibre but also 1.2-fold higher in sugar than GC bars. Both GF quickbreads and cookies were lower in protein and iron than their GC counterparts. GF quickbreads and cereal bars had 15% and 17%, respectively, less sodium than their GC counterparts.

**Table 3:** Differences in nutrient content between gluten-free and gluten-containing non-staple foods per 100 g of product, according to quickbread, cereal/granola bar, cookie and cracker sub-categorization.

	Quick breads, median (IQR)			Cereal/granola bars, median (IQR)		
Nutrient per 100 g	Gluten-free (*n* = 25)	Regular (*n* = 24)	*P* value	Gluten-free(*n* = 55)	Regular (*n* = 47)	*P* value
Calories (kcal)	300 (271–345)	307 (271–377)	0.5887	428 (393–475)	400 (378–450)	0.1836
Fat (g)	10.6 (8.2–14.7)	12.2 (10.0–17.0)	0.1544	16.3 (10.7–27.1)	10.7 (8.2–17.9)	0.0066
Saturated fat (g)	2.1 (1.3–2.9)	2.4 (2.1–3.6)	0.0488	3.1 (1.5–6.7)	4.3 (1.4–7.1)	0.6573
Carbohydrate (g)	47.1 (45.9–51.1)	46.7 (40.4–49.2)	NS	55 (47.9–71.4)	67.6 (53.6–71.4)	0.2396
Fibre (g)	1.4 (1.2–2.9)	1.6 (1.4–2.8)	0.0631	7.5 (5.9–9.5)	5.7 (3.6–7.5)	0.0026
Sugars (g)	11.5 (7.1–25.3)	18.6 (7.1–24.8)	0.8649	32.4 (22.5–36.8)	26.9 (20.0–33.9)	0.0234
Protein (g)	4.0 (2.9–5.3)	5.7 (5.5–6.3)	0.0024	8.9 (6.3–12.5)	8.6 (5.7–21.4)	0.5122
Sodium (mg)	400 (329–471)	468 (408–529)	0.0219	150 (37.5–265)	233 (200–308)	0.0003
Iron (mg)	Lower 0.8 (0.7–1.3)	2.0 (1.9–3.0)	<0.0001	2.0 (1.4–2.8)	2.4 (1.6–2.8)	0.2689

	Cookies, median (IQR)			Crackers, median (IQR)		
Nutrient per 100 g	Gluten-free (*n* = 49)	Regular (*n* = 66)	*P* value	Gluten-free (*n* = 67)	Regular (*n* = 88)	*P* value
Calories (kcal)	467 (433–485)	478 (462–485)	0.4276	450 (400–473)	450 (400–500)	0.7830
Fat (g)	20 (17.7–23.3)	20 (14.3–23.3)	0.2412	15 (5.3–22.5)	15 (6.5–19.4)	0.7148
Saturated fat (g)	9.7 (7.6–11.7)	6.3 (3.4–10.9)	0.0119	2.0 (0.5–9.1)	2.3 (0.5–7.8)	0.5396
Carbohydrate (g)	69.0 (61.5–74.2)	69.1 (65.4–75.0)	0.4916	73.3 (66.7–80.0)	68.4 (66.7–75.0)	0.0001
Fibre (g)	3.2 (0.0–3.6)	3.1 (0.0–3.6)	0.5783	5.0 (0.0–5.6)	5.0 (0.0–5.6)	0.9829
Sugars (g)	34.5 (29.0–36.7)	33.3 (25.8–38.2)	0.9865	5.0 (0.0–5.6)	6.7 (0.0–11)	<0.0001
Protein (g)	3.3 (3.2–3.8)	5.8 (4.0–6.7)	<0.0001	5.3 (5.0–10.0)	10.0 (7.1–11.1)	<0.0001
Sodium (mg)	306 (200–357)	293 (233–364)	0.8453	1.4 (1.4–1.9)	4.2 (2.0–4.7)	<0.0001
Iron (mg)	1.08 (0.93–2.49)	2.71 (1.87–3.65)	<0.0001	450 (400–473)	450 (400–500)	0.7830

**Notes.**

IQR interquartile range

### Fibre sources

Using Health Canada’s nutrient claim criteria for fibre content (Canadian Food Inspection Agency, 2018), 13.7% of GF and 25.7% of GC staple foods were high or very high in fibre, while 37.0% of GF and 43.5% of GC staples were not a source of fibre (Table S1). The greatest proportions of high fibre foods were observed in GC pastas (64.0%), GC cereals (45.0%), and GF flours (38.5%). The greatest proportions of foods not having a source of fibre were GC and GF crackers (88.6 and 79.1%, respectively) and GC flours (54.5%).

### Iron sources

Overall, 15.8% of GF and 46.9% of GC staple foods were good or very good sources of iron, while 55.6% of GF and 13.5% of GC were not considered a source of iron (Table S2). Categories with the greatest proportions of good iron sources included GC pastas (94.0%) and GC cereals (78.3%). The greatest proportions of foods not having a source of iron were GF crackers (95.5%), GC crackers (54.5%), GF breads (51.4%) and GF pastas (50.9%).

### Folate sources

Overall, 35.6% of GF and 72.2% of GC staple foods were good or very good sources of folate, while 31.1% of GF and 1.5% of GC were not considered a source of folate (Table S3).

## Discussion

To the best of our knowledge, this study presents the largest comparative nutrient analysis of packaged Canadian GF food products and their GC equivalents published to date. In agreement with previous findings around the globe, staple GF foods were higher in fat content (Missbach et al., 2015) but lower in protein (Estevez et al., 2016; Kulai & Rashid, 2014; Missbach et al., 2015), iron and folate content (Kulai & Rashid, 2014) than GC staple foods. GF pastas had less fibre content then GC counterparts, in agreement with a previous Canadian study (Kulai & Rashid, 2014). Similary, GF non-staples tended to have less micronutrients, and, in some cases, more fat and/or sugar than GC non-staples. Thus, despite the recent market growth in GF products in Canada, nutritional deficits appear to persist, making GF products not equivalent substitutes for GC products in terms of nutrient content.

Due to GF items commonly having higher fat and sugar content than their GC matches, it can be hypothesized that people who rely on processed GF foods to maintain a GF diet could be consuming calories from fat and sugar that could be higher than the levels recommended for a healthy diet. The lower protein content observed the lower protein content observed is not necessarily concerning, since grains are not key contributors to dietary protein intake. To illustrate, an Australian diet study of 105 patients with CD reported that mean protein intakes were adequate and actually higher than the general Australian population (Shepherd & Gibson, 2013). This finding was consistent with another study from the United Kingdom reporting greater mean protein intakes among 62 women on GF diets when compared to other women’s study cohorts (Wild et al., 2010). Thus, protein-rich foods may compensate for limited grain and cereal choices on the gluten-free diet, suggesting protein sufficiency.

Achieving adequate fibre intake (25 or 38 g/day for men and women, respectively) may be difficult on the GF diet, as observed with Western diets, in general (Health Canada, 2012; McGill, Fulgoni & Devareddy, 2015). Three diet studies in adults eating GF, but not children (Alzaben et al., 2015), reported inadequate fibre intake (Martin et al., 2013; Shepherd & Gibson, 2013; Wild et al., 2010). In the present study, although median fibre content of staples foods was similar between GF and GC categories (with the exception of pasta), a higher proportion of GC items were categorized as high or very high in fibre. Thus, it may be more difficult to meet fibre recommendations when following a GF diet in Canada. Interestingly, in the Austrian GF database study (Missbach et al., 2015), GF pastas had more fibre than GC pastas with no overall difference in fibre content for all foods, emphasizing the importance of country-specific GF food data.

Among whole-grain staple items, fibre was higher in GC products than in GF, likely due to the high fibre content of wheat bran. In contrast, among refined (non-whole-grain) products, fibre was greater in GF than GC items. Thus, non-whole-grain items in a GF diet should not automatically be perceived as an unhealthy item, but should be taken into account in a well planned GF diet. Secondary ingredients such as oats, brown rice flour, legume-based flours, buckwheat, quinoa and amaranth, added to GF foods, may explain these differences. Industry selection of nutritionally-dense GF flours (rather than starches), may significantly improve the fibre content of GF products.

Beyond fibre content, it is noteworthy that GF and GC flours had similar nutrient content, on average. Therefore, it is possible that preparing homemade breads and baked products with these alternative flours could improve nutrient density of a GF diet, as well as allow for control of the fat and sugar content. The challenge in using alternative flours *in natura* in food preparation is the need of high food literacy (e.g., food skills, budgeting and nutrition knowledge) and more time for meal planning and cooking in comparison to purchasing ready-to-serve products.

Micronutrients, especially iron and folate, appear to be lacking in processed GF products. The absence of mandatory iron fortification for GF grain products in Canada may explain the very low proportion of GF pastas, breads, and cereals deemed ‘good’ or ‘very good’ sources of iron, particularly when compared to their GC counterparts. The literature suggests that micronutrient fortification is inconsistent across products and brands, as well as nations (Estevez et al., 2016). No differences in iron content were reported between GF and GC food categories in Austria (Missbach et al., 2015), for example. This findings is expected, since Austria does not have mandatory fortification legislation for wheat, maize or rice (Food Fortification Initiative, 2018). Given the high prevalence of folate deficiency, iron deficiency, and anemia upon CD diagnoses (Silvester & Rashid, 2007), particular attention to iron and folate status and intake among clients with CD is warranted. The addition of folate content to the Nutrition Fact Panel in Canada would also assist in this respect, as few products appear to analyze and report folate values.

These findings provide a comprehensive insight into the GF landscape of eastern Canada. Although our ability to generalize to other regions is limited, the nutritional content of the foods reported would be consistent across Canada due to federal regulations for nutrition packaging and fortification. Due to expected differences in nutrient fortification levels, staple food items, and food formulations across countries, similar databases in other coutries would be beneficial in assessing the global industry of GF products. Even though some seasonal or out-of-stock items may have been missed from our database (for it includes only food products on display at the time of the store visits), these would be few items in comparison to the robust sample obtained. The data saturation (i.e., over 50% of the products newly sampled were duplicates) and the large sample size further mitigate these limitations. Finally, it is important to acknowledge that, by including samples only with GF labels, naturally GF raw ingredients, such as rice, fresh meat, fruits, and vegetables, were excluded. Thus, the extent of reliance on processed GF foods versus naturally GF whole foods, will have implications for the overall diet and nutritional adequacy.

## Conclusions

In summary, GF-labelled staple foods in Canada are generally less nutritious than their GC counterparts, which, together with their higher cost (Jamieson & Gougeon, 2017), further supports the notion that GF diets should not be promoted to those who do not require it (Wu et al., 2015). Notably, GF and GC flours in Canada had comparable nutritient content, suggesting that baking from scratch can offer higher nutrient density than relying on ready-to-eat GF baked products. Home baking, however, requires advanced food literacy, which includes food and nutrition knowledge, planning, budgeting, and food skills. Evaluation of GF diets should consider the use of packaged GF foods versus homemade or whole food use and ensure the appropriate replacement of wheat in the diet with complementary nutrient-rich grains.

##  Supplemental Information

10.7717/peerj.5875/supp-1 Data S1 Canadian Gluten-free product food database

10.7717/peerj.5875/supp-2 Table S1 Fibre content claims of gluten-free and regular gluten-containing foods

10.7717/peerj.5875/supp-3 Table S2 Iron content claims of staple gluten-free and regular gluten-containing foods

10.7717/peerj.5875/supp-4 Table S3 Folate content claims of staple gluten-free and regular gluten-containing foods

## Additional Information and Declarations

### Data Availability

The following information was supplied regarding data availability: The raw data are provided in the Supplemental Files.
