# Supplementary material for: Canadian packaged gluten-free foods are less nutritious than their regular gluten-containing counterparts
Source: PeerJ. 2018 Nov 6;6:e5875. doi: 10.7717/peerj.5875 (PMC6225834; doi:10.7717/peerj.5875)
Supplement: Table S1 [file peerj-06-5875-s002.docx]

Table S2.

|  |  | Not a source  (< 2 g/serving) | | Source  (≥2 to <4 g/serving) | | High in fibre  (≥4 to <6 g/serving) | | Very high  (≥6 g/serving) | |
| --- | --- | --- | --- | --- | --- | --- | --- | --- | --- |
|  |  | n | % | n | % | n | % | n | % |
| Breads | GF n=72 | 23 | 31.9 | 29 | 40.3 | 10 | 13.9 | 10 | 13.9 |
|  | GC n=87 | 30 | 34.5 | 43 | 49.4 | 11 | 12.6 | 3 | 3.5 |
| Flours | GF n=26 | 6 | 23.1 | 10 | 38.5 | 8 | 30.8 | 2 | 7.7 |
|  | GC n=25 | 12 | 48.0 | 10 | 40.0 | 5 | 20.0 | 0 | 0.0 |
| Cereals | GF n=52 | 12 | 23.1 | 23 | 44.2 | 13 | 25.0 | 4 | 7.7 |
|  | GC n=60 | 14 | 23.3 | 19 | 31.7 | 13 | 21.7 | 14 | 23.3 |
| Pastas | GF n=53 | 7 | 13.2 | 44 | 83.0 | 2 | 3.8 | 0 | 0.0 |
|  | GC n=50 | 1 | 2.0 | 17 | 34.0 | 5 | 10.0 | 27 | 54.0 |
|  | | | | | | | | | |
| Total Staples | GF n=202 | 48 | 23.8 | 106 | 52.5 | 32 | 15.8 | 16 | 7.9 |
|  | GC n=220 | 57 | 25.9 | 84 | 38.2 | 35 | 15.9 | 44 | 20.0 |
|  | | | | | | | | | |
| Total Non-staples | GF n=196 | 122 | 62.2 | 48 | 24.5 | 22 | 11.2 | 4 | 2.1 |
|  | GC n=225 | 161 | 71.6 | 47 | 20.9 | 15 | 6.7 | 2 | 0.9 |
