# Supplementary material for: Canadian packaged gluten-free foods are less nutritious than their regular gluten-containing counterparts
Source: PeerJ. 2018 Nov 6;6:e5875. doi: 10.7717/peerj.5875 (PMC6225834; doi:10.7717/peerj.5875)
Supplement: Table S2 [file peerj-06-5875-s003.docx]

Table S3.

| Category |  | Not a source  (< 5 % RDI) | | Source (≥5 to <15 % RDI) | | Good source (≥15 to <25 % RDI) | | Very good/Excellent source (≥25 % RDI) | |
| --- | --- | --- | --- | --- | --- | --- | --- | --- | --- |
|  |  | n | % | n | % | n | % | n | % |
| Breads | GF n=72 | 37 | 51.4 | 28 | 48.6 | 7 | 9.7 | 0 |  |
|  | GC n=87 | 0 | 0.0 | 87 | 100 | 38 | 43.7 | 7 | 8.0 |
| Flours | GF n=25 | 8 | 32.0 | 10 | 40.0 | 2 | 8.0 | 5 | 20.0 |
|  | GC n=23 | 0 | 0.0 | 21 | 91.3 | 1 | 4.4 | 1 | 4.4 |
| Cereals | GF n=52 | 14 | 26.9 | 24 | 46.2 | 8 | 15.4 | 6 | 11.5 |
|  | GC n=60 | 1 | 1.7 | 12 | 20.0 | 5 | 8.3 | 42 | 70.0 |
| Pastas | GF n=53 | 27 | 50.9 | 22 | 41.5 | 1 | 1.9 | 3 | 5.7 |
|  | GC n=50 | 1 | 2.0 | 2 | 4.0 | 40 | 80.0 | 7 | 14.0 |
|  | | | | | | | | | |
| Total Staples | GF n=202 | 86 | 42.6 | 84 | 41.6 | 18 | 8.9 | 14 | 6.9 |
|  | GC n=220 | 2 | 0.91 | 77 | 35.0 | 84 | 38.2 | 57 | 25.9 |
|  | | | | | | | | | |
| Total Non-staples | GF n=96 | 140 | 71.4 | 51 | 26.0 | 4 | 2.0 | 1 | 0.5 |
|  | GC n=225 | 96 | 42.7 | 107 | 47.6 | 20 | 8.9 | 2 | 0.9 |

RDI = recommended daily intake (14 mg iron/day)
