# Supplementary material for: Canadian packaged gluten-free foods are less nutritious than their regular gluten-containing counterparts
Source: PeerJ. 2018 Nov 6;6:e5875. doi: 10.7717/peerj.5875 (PMC6225834; doi:10.7717/peerj.5875)
Supplement: Table S3 [file peerj-06-5875-s004.docx]

Table S4.

|  |  | Not a source  (< 5 % RDI) | | Source (≥5 % RDI) | | Good source (≥15 % RDI) | | Very good/Excellent source (≥25 % RDI) | |
| --- | --- | --- | --- | --- | --- | --- | --- | --- | --- |
|  |  | n | % | n | % | n | % | n | % |
| Staples | GF 45 | 14/45 | 31.1 | 31/45 | 68.9 | 16/45 | 35.6 | 6/45 | 13.3 |
|  | GC 133 | 2/133 | 1.5 | 131/133 | 98.5 | 96/133 | 72.2 | 49/133 | 36.8 |
|  |  |  |  |  |  |  |  |  |  |

RDI = recommended daily intake (220 µg folate/day)
